# 3D-printed phantom study for investigating stent abutment during gastroduodenal stent placement for gastric outlet obstruction

**DOI:** 10.1186/s41205-017-0017-0

**Published:** 2017-09-25

**Authors:** Guk Bae Kim, Jung-Hoon Park, Ho-Young Song, Namkug Kim, Hyun Kyung Song, Min Tae Kim, Kun Yung Kim, Jiaywei Tsauo, Eun Jung Jun, Do Hoon Kim, Gin Hyug Lee

**Affiliations:** 1 Biomedical Engineering Research Center, Asan Medical Center, University of Ulsan College of Medicine, 388-1, Poongnap 2-dong, Songpa-gu, Seoul, Republic of Korea; 2 Radiology and Research Institute of Radiology, Asan Medical Center, University of Ulsan College of Medicine, 388-1, Poongnap 2-dong, Songpa-gu, Seoul, Republic of Korea; 3 Department of Convergence Medicine, Asan Medical Center, University of Ulsan College of Medicine, 388-1, Poongnap 2-dong, Songpa-gu, Seoul, Republic of Korea; 4 Gastroenterology, Asan Medical Center, University of Ulsan College of Medicine, 388-1, Poongnap 2-dong, Songpa-gu, Seoul, Republic of Korea; 5 Department of Radiology and Convergence Medicine, Asan Medical Center, University of Ulsan College of Medicine, 388-1, Poongnap 2-dong, Songpa-gu, Seoul, 138-736 Republic of Korea; 6 Department of Radiology, Asan Medical Center, University of Ulsan College of Medicine, 388-1, Poongnap 2-dong, Songpa-gu, Seoul, 138-736 Republic of Korea

**Keywords:** 3D Printing, Self-expandable metallic stent, Stent abutment, Gastroduodenal phantom

## Abstract

**Background:**

Placing a self-expandable metallic stent (SEMS) is safe and effective for the palliative treatment of malignant gastroduodenal (GD) strictures. SEMS abutment in the duodenal wall is associated with increased food impaction, resulting in higher stent malfunction and shorter stent patency. The desire to evaluate the mechanism and significance of stent abutment led us to design an in vitro experiment using a flexible anthropomorphic three-dimensional (3D)-printed GD phantom model.

**Results:**

A GD phantom was fabricated using 3D printer data after performing computed tomography gastrography. A partially covered (PC) or fully covered (FC) stent was placed so that its distal end abutted onto the duodenal wall in groups PC-1 and FC-1 or its distal end was sufficiently directed caudally in groups PC-2 and FC-2. The elapsed times of the inflowing of three diets (liquid, soft, and solid) were measured in the GD phantom under fluoroscopic guidance. There was no significant difference in the mean elapsed times for the liquid diet among the four groups. For the soft diet, the mean elapsed times in groups PC-1 and FC-1 were longer than those in groups PC-2 and FC-2 (*P* = 0.018 and *P* < 0.001, respectively). For the solid diet, the mean elapsed time in group PC-1 was longer than that in group PC-2 (*P* < 0.001). The solid diet could not pass in group FC-1 due to food impaction. The mean elapsed times were significantly longer in groups FC-1 and FC-2 than in groups PC-1 and PC-2 for soft and solid diets (all *P* < 0.001).

**Conclusions:**

This flexible anthropomorphic 3D–printed GD phantom study revealed that stent abutment can cause prolonged passage of soft and solid diets through the stent as well as impaction of solid diets into the stent.

## Background

The placement of a partially covered (PC) or fully covered (FC) self-expandable metallic stent (SEMS) is safe, easy, and effective for the palliative treatment of malignant gastroduodenal (GD) strictures [1–7]. Their overall technical and clinical success rates have been reported to be 94 to 100% and 94 to 94.8%, respectively, in studies that have included >74 but <213 patients [3–7]. Recently, our experience with the placement of a SEMS bridging the gastric pylorus for gastric outlet obstruction (GOO) caused by inoperable gastric cancer showed that the distal end of the stent abutted onto the duodenal wall in 107 (33.6%) of 318 patients. Further, stent abutment was associated with increased food impaction, resulting in higher stent malfunction and shorter stent patency [8].

However, the study was a retrospective analysis and was not sufficient to reveal the mechanism of food impaction related with stent abutment. Moreover, only a PC stent was used. The desire to evaluate the mechanism and significance of stent abutment in the duodenal wall led us to design an in vitro experiment using a flexible anthropomorphic three-dimensional (3D)-printed GD phantom model. Recently, 3D printing (3DP) has been commonly used in various fields of medicine, including for personalized treatment, medical research, and premedical education, for soft and hard tissues [9–12]. Therefore, the purpose of this study was to investigate the significance of stent abutment in food passage in comparison with non-stent abutment using an anthropomorphic 3D–printed GD phantom model.

## Methods

### Design of the 3D–printed GD phantom model

Computed tomography gastrography (CTG) findings of a patient (62-year-old man with advanced gastric cancer involving the gastric pyloric antrum) were retrospectively used to construct an anthropomorphic 3D–printed GD phantom model. The patient had fasted for at least 8 h prior to undergoing CTG. In total, 10 mg of butylscopolamine (Buscopan, Boehringer Ingelheim, Seoul, Korea) was intravenously injected to decrease bowel peristalsis. The patient received 6 g of effervescent granules (Taejeon Pharmaceuticals, Kyungki-Do, Korea) with 10 ml of water to achieve gastric distension prior to CT. Triphasic CT scans (Somatom Sensation 16, Siemens, Erlangen, Germany) were performed during the arterial phase (start of delay, 30 s) with the patient in the left posterior oblique (LPO) position, during the portal phase (72 s) with the patient in the supine position, and during the delayed phase (150 s) with the patient in the prone position after the injection of 120 ml of a nonionic contrast material (Ultravist, Schering, Berlin, Germany) at 4 ml/s via the antecubital vein using an 18-gauge needle and an automatic injector. The LPO position was maintained by placing a pillow below the patient’s back [13].

**Fig. 1:**
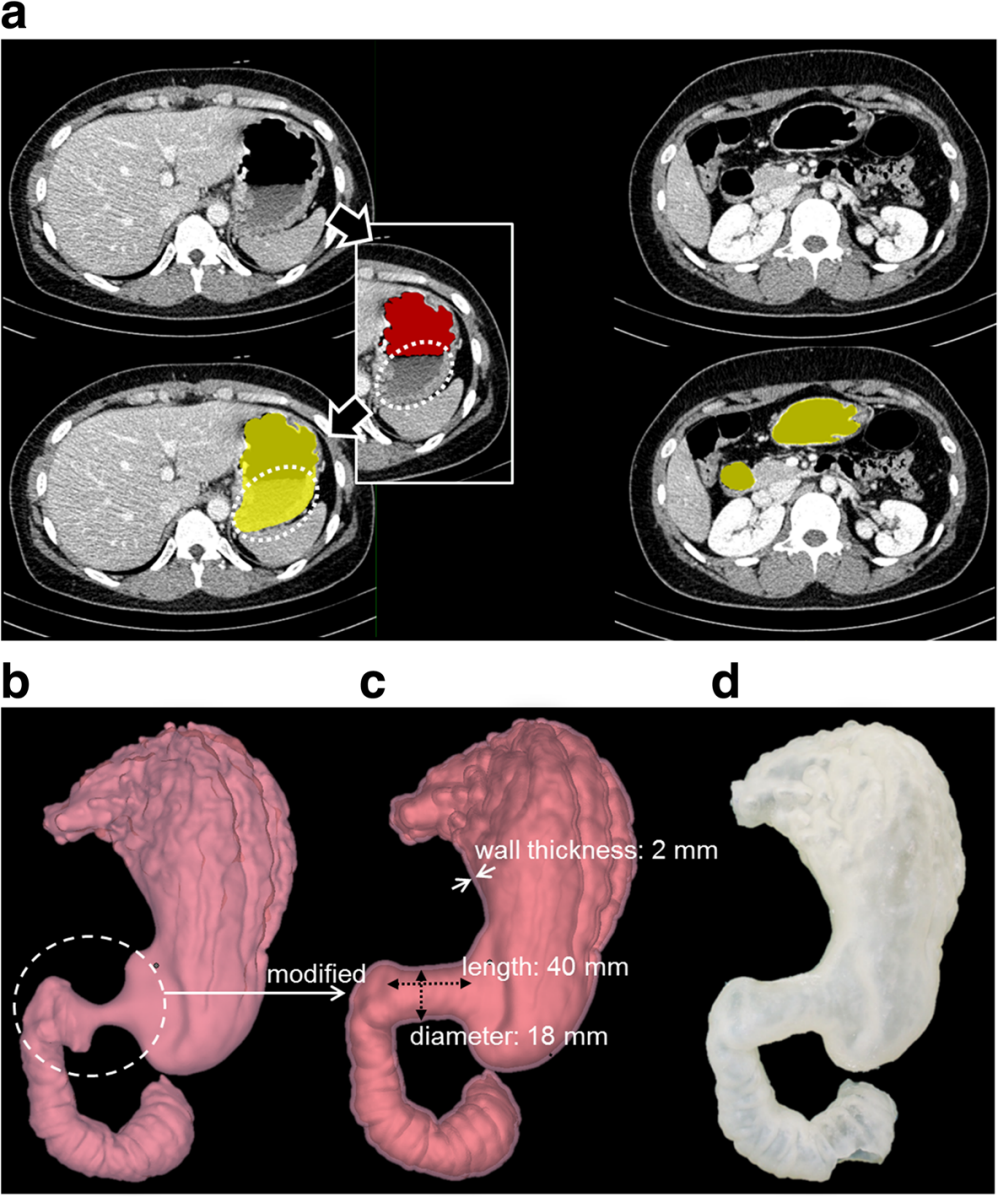
Process for preparing the 3D–printed GD phantom from CTG data. **a** CT images with segmentation masks. A red mask was made using a simple thresholding setting. Yellow masks were the final segmentation result after multi-step imaging. **b** Initial 3D GD digital model. **c** Modified 3D GD digital model for stent placement. **d** Anthropomorphic 3D–printed GD phantom model

CT image data were imported to the in-house advanced software (AVIEW, Asan Medical Center, Seoul, Korea) to obtain a 3D visualization model of the entire stomach and duodenum, which was saved in the Stereolithography (STL) file format. Based on the 3D reconstructed models, the length (40 mm) and diameter (18 mm) of the gastric outlet were modified according to stent size using two open source programs (MeshLab and MeshMixer) because the 3D printable material was not flexible enough to stretch from in vivo dimensions. Degree of thickness of the model wall was generated by 2 mm. The modified 3D visualization model was saved in the G-code format and exported to a 3D printer (Objet500 Connex3, Stratasys Corporation, Rehovot, Israel). The 3D–printed GD phantom was made of 100% rubber-like material (Tango™ Family) (Fig. 1).

### Stents and placement

**Fig. 2:**
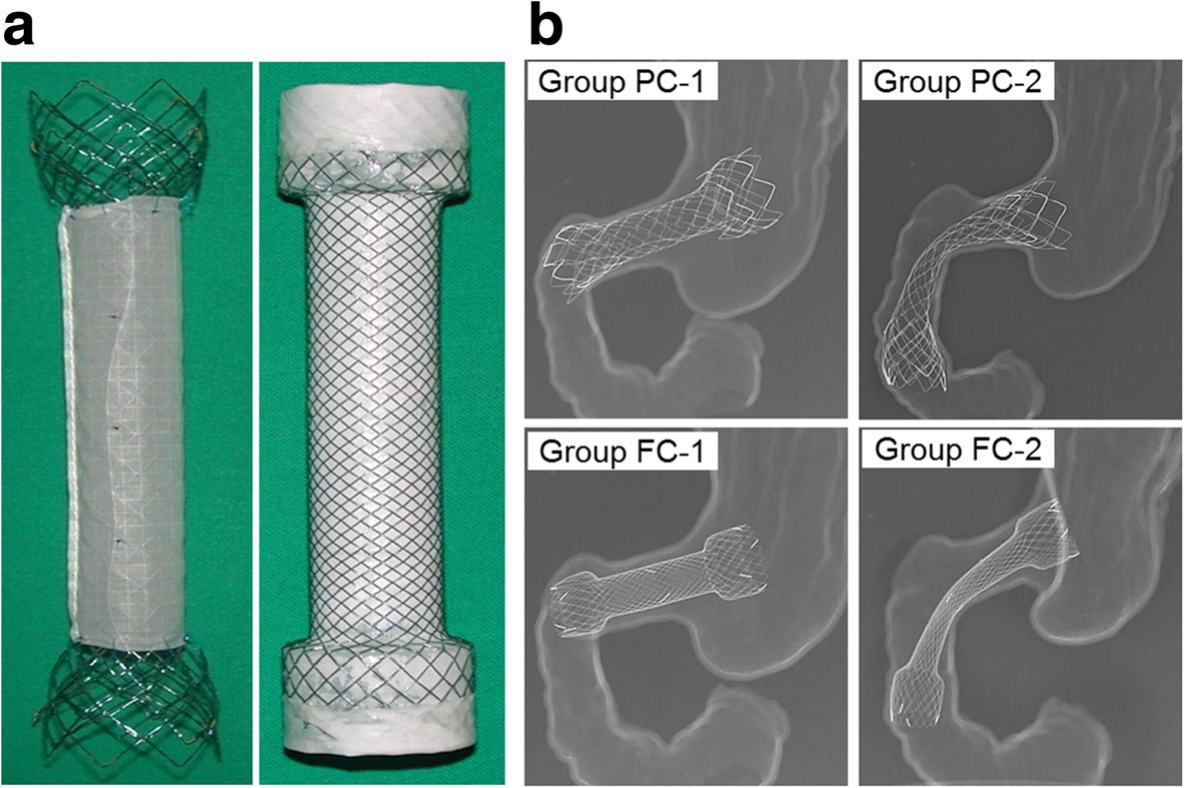
Two types of SEMSs. **a** PC stent (left) and FC stent (right). **b** Radiographs of the four groups. Groups PC-1 and Group FC-1 indicate the PC and FC stents with abutment in the duodenal wall, respectively. Groups PC-2 and Group FC-2 indicate the PC and FC stents without abutment, respectively

Two types of SEMS were used: a PC stent (S&G Biotech, Seongnam, Korea) and a FC stent (Taewoong Medical, Gimpo, Korea) (Fig. 2). The PC stent consisted of two stents: an outer, partially covered stent and an inner, bare nitinol stent. The inner and outer stents were 18 mm in diameter, with their ends flared up to 28 mm. Both ends of the FC stent were 24 mm in diameter and 20 mm in length when fully expanded, and its body was 18 mm in diameter. A detailed description of the stent and delivery systems with their placement techniques has been previously reported [3, 4].

The stents were divided into four groups according to the types of stents and the location of their distal ends. PC and FC stents were placed so that their distal ends abutted the duodenal wall: groups PC-1 and FC-1, respectively. Conversely, PC and FC stents were placed so that their distal ends were sufficiently directed caudally to prevent stent abutment: groups PC-2 and FC-2, respectively.

### Food materials

Three different kinds of radiopaque food materials that varied in their degree of thickness were prepared. Telebrix (30 meglumine, 300 mg/ml; Guerbet, Gorinchem, the Netherlands) was used as the liquid diet. A mixture of yogurt and barium sulfate (98% *w*/w; E-Z-HD; E-Z-EM Canada Inc., NJ, Canada) in a 3:1 ratio and a mixture of congee and barium sulfate in a 3:1 ratio were used as the soft and solid diets, respectively.

### Experiments

**Fig. 3:**
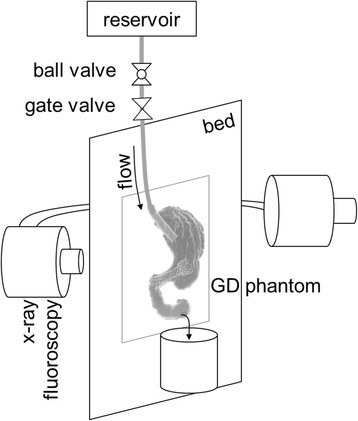
Schematic image of the experimental setup under fluoroscopic guidance: reservoir, two valves, the GD phantom fixed in a foamed polystyrene box, and an exit beaker

Figure 3 shows the experimental setup in the GD phantom. The prepared GD phantom was fixed in an expanded polystyrene foam box, and a silicone tube was connected to the inlet of the GD phantom for inflowing of food materials under fluoroscopic guidance. Inflowing of food materials was controlled by two-piece ball valves to control the flow rate and a general valve for inflowing on/off. Inflowing in the liquid diet was carefully controlled by the ball valve to mimic the situation of drinking water for approximately 4 s (300 ml). To quantitatively evaluate food inflowing differences, elapsed times were measured from when the food material (300 ml in volume) started into the inlet to when the food material (200 ml in volume) passed through the outlet of the GD phantom and was filled in an exit beaker. Twelve elapsed times were acquired with respect to the two conditions of stent placement, two kinds of stents, and three food materials. Each elapsed time was repeated 10 times.

### Statistical analysis

Variables were compared using Student’s unpaired *t* test. A two-sided *P*-value of <0.05 was considered to indicate statistical significance. All statistical analyses were performed using SPSS (version 21, SPSS).

## Results

**Fig. 4:**
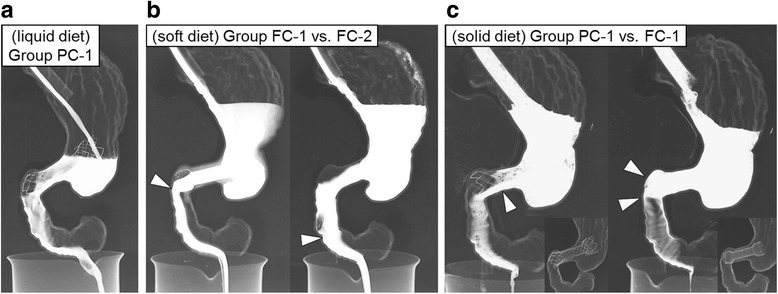
Representative radiographs of food inflowing in the GD phantom. **a** Radiograph obtained 20 s after inflowing in group PC-1 using the liquid diet shows the disturbance-free passage of radiopaque liquid diet. **b** Radiographs obtained 20 s after inflowing in groups FC-1 and FC-2 using the soft diet show the narrowing passage of the diet (arrowhead) though the distal end of the stent in group FC-1 and relatively better passage of the diet (arrowhead) though the distal end of the stent in group FC-2. **c** Radiographs obtained 20 s after inflowing in groups PC-1 and FC-1 using the solid diet show poor passage of the diet through the wire mesh of the stent (arrowhead) in group PC-1 and food impaction (arrowheads) in group FC-1

The mean elapsed times for the liquid diet were not significantly different among the four groups (4.21 ± 0.04 s in group PC-1, 4.22 ± 0.04 s in group FC-1, 4.22 ± 0.05 s in group PC-2, and 4.23 ± 0.04 s in group FC-2). For the soft diet, the mean elapsed times were longer in groups PC-1 (7.00 ± 0.09 s) and FC-1 (9.06 ± 0.25 s) than in groups PC-2 (6.88 ± 0.10 s) and FC-2 (8.32 ± 0.10 s), with a statistically significant difference between the stent types (*P* = 0.018 and *P* < 0.001, respectively). For the solid diet, the mean elapsed times were longer in group PC-1 (32.35 ± 1.10 s) than in group PC-2 (24.53 ± 0.37 s) (*P* < 0.001). The mean elapsed time was 42.78 ± 1.33 s in group FC-2; conversely, in group FC-1, the solid diet could not pass through the FC stent because food impaction occurred immediately (Fig. 4).

The mean elapsed times in groups FC-1 and FC-2 was significantly longer than in groups PC-1 and PC-2 for the soft and solid diets (all P < 0.001). The mean elapsed times are summarized in Tables 1 and 2.

**Table 1:** Mean elapsed times between the stent abutment and non-stent abutment groups

Diet type	Partially Covered Stent			Fully Covered Stent		
	PC-1	PC-2	*P*-value	FC-1	FC-2	*P*-value
Liquid	4.21 ± 0.04	4.22 ± 0.05	0.625	4.22 ± 0.04	4.23 ± 0.03	0.609
Soft	7.00 ± 0.09	6.88 ± 0.10	0.018	9.06 ± 0.25	8.32 ± 0.07	<0.001
Solid	32.35 ± 1.10	24.53 ± 0.38	<0.001	FI	42.78 ± 1.33	NA

*Note*. Unit of data is seconds; *FI* Food impactiong, *NA* not applicable

**Table 2:** Mean elapsed times between the partially and fully covered stent groups

Diet type	Stent abutment condition			Non-stent abutment condition		
	PC-1	FC-1	*P*-value	PC-2	FC-2	*P*-value
Liquid	4.21 ± 0.04	4.22 ± 0.04	0.630	4.22 ± 0.05	4.23 ± 0.03	0.714
Soft	7.00 ± 0.09	9.06 ± 0.25	<0.001	6.88 ± 0.10	8.32 ± 0.07	<0.001
Solid	32.35 ± 1.10	FI	NA	24.53 ± 0.38	42.78 ± 1.33	<0.001

*Note*. Unit of data is seconds; *FI* Food impactiong, *NA* not applicable

## Discussion

It is still not known how food materials move down the GD pathway according to SEMS locations and stent types (PC or FC). Park et al. recently reported that patients with stent abutment were more likely to have food impaction than those without stent abutment, resulting in higher rates of stent malfunction and shorter stent patency durations [8]. They indicated that the distal landing zone of the stent should be located in the second portion of the duodenum to prevent stent abutment. The concept of stent abutment may be useful for avoiding stent malfunction, particularly from food impaction. An experimental study that quantitatively explains food inflowing differences according to stent abutment, even using an artificial GD phantom model, is required.

The results of the present study demonstrated that the mean elapsed times in the passage of a soft and solid diet were significantly longer in the stent abutment groups (PC-1 and FC-1) than in the non-stent abutment groups (PC-2 and FC-2) and that the mean elapsed times were statistically longer with FC stents than with PC stents. Mention The results of our experimental study correlated with clinically significant findings of stent abutment in patients with GOO [8].

The GOO scoring system [14] the Song dysphagia classification [3] was routinely used to evaluate the severity of dysphagia before and after stent placement. The scores were based on the ability to swallow liquid, soft, and solid diets [3, 14]. To establish radiopaque liquid, soft, and solid diets, a water-soluble contrast medium and barium sulfate with a mixture of yogurt and congee were used in the present study. The elapsed times using these diets were investigated according to the stent abutment with two different types of SEMS. The results of our experimental study under fluoroscope guidance showed different elapsed times according to the food materials.

In the representative radiographs (Fig. 4), a typical liquid diet showed inflowing without any disturbance in the experimental setup. For soft diets with the FC stent, the non-stent abutment group showed better inflowing performance at the end of the stent than the stent abutment group (white arrow heads in Fig. 4b). Figure 4c shows food impaction found in the FC stent group under stent abutment for the solid diet (white arrow heads on the right). Conversely, in the PC stent group, diet inflowing in the GD phantom was very narrow but food impaction did not occur under the same stent abutment conditions (white arrow head on the left). We believe that the position of the distal ends of the stent is very important during stent placement in patients with GOO.

3D reconstructed images are not only pivotal in making a diagnosis but also essential in the advent of 3DP in the medical field using a tangibly fabricated 3D model [15, 16]. With the recent medical trend toward “personalized” or “patient-specific” treatment, 3DP has well-suited characteristics in the field of medicine [16]. It can perform one-stop manufacturing from radiological images to patient-specific phantoms, simulators, surgical guides, or implantable devices [17–21]. The recently commercialized 3D printable multi-materials with transparent, full-colored, and flexible properties accelerate its applications to more extensive medical fields [16, 22–24]. In the present study, a patient-specific and anthropomorphic GD phantom was fabricated using 3DP based on CTG images. However, the GOO region of the initial GD STL model was modified to fit the stent size.

We attempted to mimic GD food inflowing with the maximum amount of effort in the present phantom study, but it is still far from reality. Firstly, the 3D printable material used is flexible but not enough to mimic real GD properties due to their technical limitations. One could also utilize a 3D printing-based molding method using silicone materials whose mechanical properties are more controllable. The mechanical properties of GD tissue, such as shore hardness and elongation, also need to be determined. Secondly, we need to materialize gastric peristalsis with a motorized system for obtaining more realistic digesting situations. With these methodological advances, it is expected that more realistic GD situations can be mimicked in future experimental phantom studies.

## Conclusions

The findings of the present study revealed that stent abutment can cause prolonged passage of soft and solid diets through the stent and can also cause impaction of the solid diet into the stent. Therefore, we believe that when a stent is placed in the gastric outlet bridging the gastric pylorus, the distal end of the stent should be directed caudally to prevent stent abutment.

## Abbreviations

3D: Three-dimensional
3DP: 3D printing
CTG: Computed tomography gastrography
FC: Fully covered
GD: Gastroduodenal
GOO: Gastric outlet obstruction
LPO: Left posterior oblique
PC: Partially covered
SEMS: Self-expandable metallic stent
STL: Stereolithography
